# Bibliometric assessment and key messages of sporotrichosis research (1945-2018)

**DOI:** 10.12688/f1000research.24250.2

**Published:** 2021-04-09

**Authors:** Priscila Costa Albuquerque, Bruna de Paula Fonseca e Fonseca, Fabio Zicker, Rosely Maria Zancopé-Oliveira, Rodrigo Almeida-Paes

**Affiliations:** 1 Center for Technological Development in Health (CDTS), Oswaldo Cruz Foundation, Rio de Janeiro, RJ, 21040361, Brazil; 2 Mycology Laboratory, Evandro Chagas National Institute of Infectious Diseases (INI), Oswaldo Cruz Foundation, Rio de Janeiro, RJ, 21040900, Brazil

**Keywords:** Bibliometrics, Network, Scientometry, Sporothrix, Sporotrichosis

## Abstract

**Background: **Sporotrichosis has recently emerged as an important mycosis worldwide, with diverse transmission and epidemiologic profiles. For instance, in Brazil most cases are related to zoonotic transmission from naturally infected cats, and the majority of cases in China are due to external injury with environmental materials. Publications on sporotrichosis and on its etiologic agent may guide the direction of the research in this field. It can also define priorities for future studies.

**Methods: **In this study, we evaluated the trends of global research in *Sporothrix *and sporotrichosis, based on publications records retrieved from Scopus and Web of Science databases for the period of 1945 to 2018. The overall productivity in the field, its geographical and temporal distribution, research themes, co-authorship networks, funding sources, and if audience and research findings are addressed in the abstracts.

**Results: **A total of 4,007 unique publications involving 99 countries were retrieved, most of them published after 2000. Authors based on institutions from the United States of America and Brazil accounted for 57.4% of the publications. Brazil was the leading country in terms of research collaboration and networking, with co-authorship with 45 countries. The thematic mapping revealed a temporal shift from clinical to applied research. Despite the large number of countries publishing in this field, most of funded studies came from Brazil, Mexico, China, South Africa, or the United States of America. The analysis of content identified few specific public health recommendations for prevention, case-management, or research. Moreover, most papers do not have a clearly defined intended audience.

**Conclusion: **As the research in this field is emerging in several countries, with the generation of a large amount of data, it is necessary that scientists strengthen efforts to translate the research results into practice to curb this neglected infection.

## Introduction

Sporotrichosis is a subcutaneous mycotic infection caused by dimorphic species of fungi belonging to the genus *Sporothrix* ^1^. It has a worldwide distribution, a broad range of clinical presentations, and can be fatal as an opportunistic infection in immunosuppressed patients ^2^. For more than one century, the etiological agent of sporotrichosis was identified as the sole species *Sporothrix schenckii* ^3^. However, in last years, using molecular biology techniques, it was possible to identify other sibling species that also cause sporotrichosis: *Sporothrix brasiliensis*, *Sporothrix globosa*, and, to a lesser extent, *Sporothrix luriei*, *Sporothrix pallida*, *Sporothrix mexicana*, ** and *Sporothrix chilensis* ^4– 7^. These agents can be found in the environment ^1^ and they present different clinical manifestations ^8^, virulence ^9^, drug susceptibility ^10^, and phenotypic characteristics ^11^.

The research about fungal diseases has been relatively neglected worldwide by public health authorities. The initiative “Global Action Fund for Fungal Infections” (GAFFI), an international non-governmental organization dedicated to combating fungal disease, was created to highlight gaps in diagnostics and treatments for fungal diseases as well as to fund raise and lobby global health agencies ^12^. GAFFI has identified some fungal infections as its highest priorities and sporotrichosis, along with other deep mycoses, is included ^13^. This organization has claimed that sporotrichosis, paracoccidioidomycosis, and fungal keratitis should be included in the WHO’s Neglected Tropical Diseases portfolio. This would be a big step towards increasing research funding and better care for patients with these serious diseases.

Bibliometric studies are frequently used to describe the global dynamics of knowledge generation and provide useful information on research discoveries, pointing at the strengths and weakness of new findings ^14^. As an example, through bibliometric analyses, based on quantitative and qualitative indicators, it was possible to assess the progress and collaboration in science, technology and innovation in the tuberculosis field ^15^. For instance, the co-authorship of scientific publications reveals collaborative patterns between individuals, organizations, or countries ^16^ and represents a formal statement of interaction between two or more researchers, being widely used to understand and assess collaboration profiles ^17, 18^. Bibliometric methods, however, have rarely been applied to the mycology field and not much is known about the extension and trends of *Sporothrix* research. Therefore, an assessment of the characteristics of the research focusing on sporotrichosis and its etiological agents is necessary to evaluate the progress of the findings in this field and the implications and impacts of sporotrichosis research for health practice.

Driven by the continued expansion of sporotrichosis in some countries such as Brazil, China, Mexico, and India ^1^, we analyzed the global scientific publications and scientific collaboration on the *Sporothrix* and sporotrichosis research field, with emphasis on the impact of the zoonotic epidemic of sporotrichosis in Brazil ^2^ and on the discovery of the new species genetically related to *S. schenckii* ^5^. We combined bibliometrics and social network analysis to generate evidence of the dynamics of the research community. Also, this research aims to evaluate a methodology to identify key messages and technical recommendations for action in the abstracts of the publications assessed, to check whether the research results were being translated into actions to curb sporotrichosis.

## Methods

### Source of data

Publications were retrieved from the Web of Science (WoS) and Scopus databases searching for the terms “ *Sporothrix*” or “sporotrichosis” on the title, abstract, and keyword fields. The review included original research articles, reviews, letters to the editor, and editorials from 1945 to 2018 (WoS Core Collection). For comparison purposes, the number of publications about other mycoses caused by dimorphic fungi (paracoccidioidomycosis, histoplasmosis, coccidiocomycosis, and blastomycosis) was also obtained, using the genus of the fungus and the name of the mycosis as the search query terms.

### Cleaning and standardization of data

The data retrieved was imported into the text mining software VantagePoint 10.0 (Search Technology Inc. Norcross, GA, USA) and duplicate records were excluded. Names of institutions, countries, funding organizations, and journals were standardized using the VantagePoint list cleanup tool, and further manual processing. An open-access alternative for the use of VantagePoint 10.0 is the software OpenRefine 3.3.

### Co-authorship network analyses

After cleaning and standardization, the data was formatted into adjacency matrixes to map co-authorship between countries and institutions based on authors’ professional affiliations. The matrixes were imported into the open-source software Gephi 0.9.1 for network visualization and calculation of metrics ^19^. Degree centrality was used to identify the most central institutions in the network, reflecting the significance of a network member (node) relative to all other nodes in the network. This metric takes into account the diverse means in which a node interacts and communicates with the rest of the network. The most important, or central ones, have a strategic impact in the network. The degree centrality can be explained as the number of direct links that a node has with other nodes. The more relational ties a node has, more power or prestige it may present in a network ^20^. Betweenness centrality was used to recognize organizations that mediated the connection between other institutions and their capacity to control the flow of information in the network ^20^. This metric reveals the extent to which a node works as a bridge among the other nodes in the network, which would otherwise be disconnected. For the spatial visualization of international collaboration, the authors’ professional affiliation country was manually geocoded and processed using the “ GeoLayout” 0.9.1.2 and “ Map of Countries” 1.5.1 plugins available within Gephi. In these networks, nodes represent countries or an institution, and two or more countries/institutions were connected if their researchers shared the authorship of one or more papers. As co-authorship involves reciprocal collaboration, all connections were considered as non-directional.

### Mapping research themes

Term maps were created using the VOSviewer 1.6.6 software (Leiden University, the Netherlands) using terms obtained from titles and abstracts of all publications in the database. Each term was graphically denoted by a circle whose diameter and label size were directly proportional to their frequency. The software positions the circles closer to each other according to the power of the relationship and co-occurrence between terms. The mapping allowed a cluster analysis by research themes using a weighted and parameterized variant of modularity-based clustering to recognize groups of correlated terms ^21^.

### Funding data

Funding acknowledgments on publications were only available as a searchable field in WoS and Scopus from late 2008. In order to achieve reliable coverage, only papers published from 2012 onwards were selected for this purpose. Funding agencies were identified, their names standardized (whenever possible), and the number of publications per funding agency summarized.

### Retrieving key messages from abstracts

In order to evaluate the applicability of a wording methodology to retrieve key messages, a screening was performed by two independent evaluators in the abstracts for statements or recommendations for sporotrichosis research, case-management, and prevention, searching keywords related to prescriptive, tentative or informative languages. In addition, the two evaluators also searched the audience mentioned in the abstracts. Titles and abstracts were tabulated on a Microsoft Excel 2010 spreadsheet, and searched for terms related to statements and recommendations for research, practice or public health. A set of words related to tentative language (“may”, “might”, “speculate”, “suggest”, or “potentially”), prescriptive language (“must”, “propose”, “should”, “stress”, or “recommend”) or related to minimal advice (“consider”, “advise”, “notify”, or “inform”) was used in this process ^22^. The audiences to whom the recommendations were directed to (medical doctors, nurses, laboratory staff, or veterinarians) were also identified.

### Statistical analysis

The software GraphPad Prism 5 was used to build linear regressions, to check the frequency of publications over time and to compare slopes of different best-fit lines. The Chi-square test was used to test for differences in proportions of “tentative”, “prescriptive”, and “minimal advice” from research results according to the country of origin in an attempt to identify how explicit was the research message to the scientific community, practitioners and public health professionals. A *p*<0.05 was considered to be significant.

## Results

### The scientific literature on *Sporothrix* and sporotrichosis

**Figure 1.**
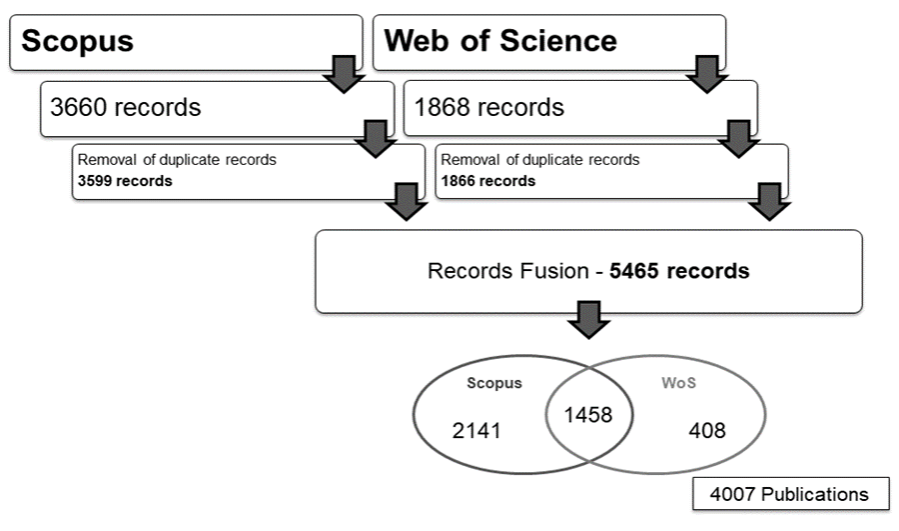
Process of publication acquisition in the *Sporothrix*/sporotrichosis field from the studied databases.

**Figure 2.**
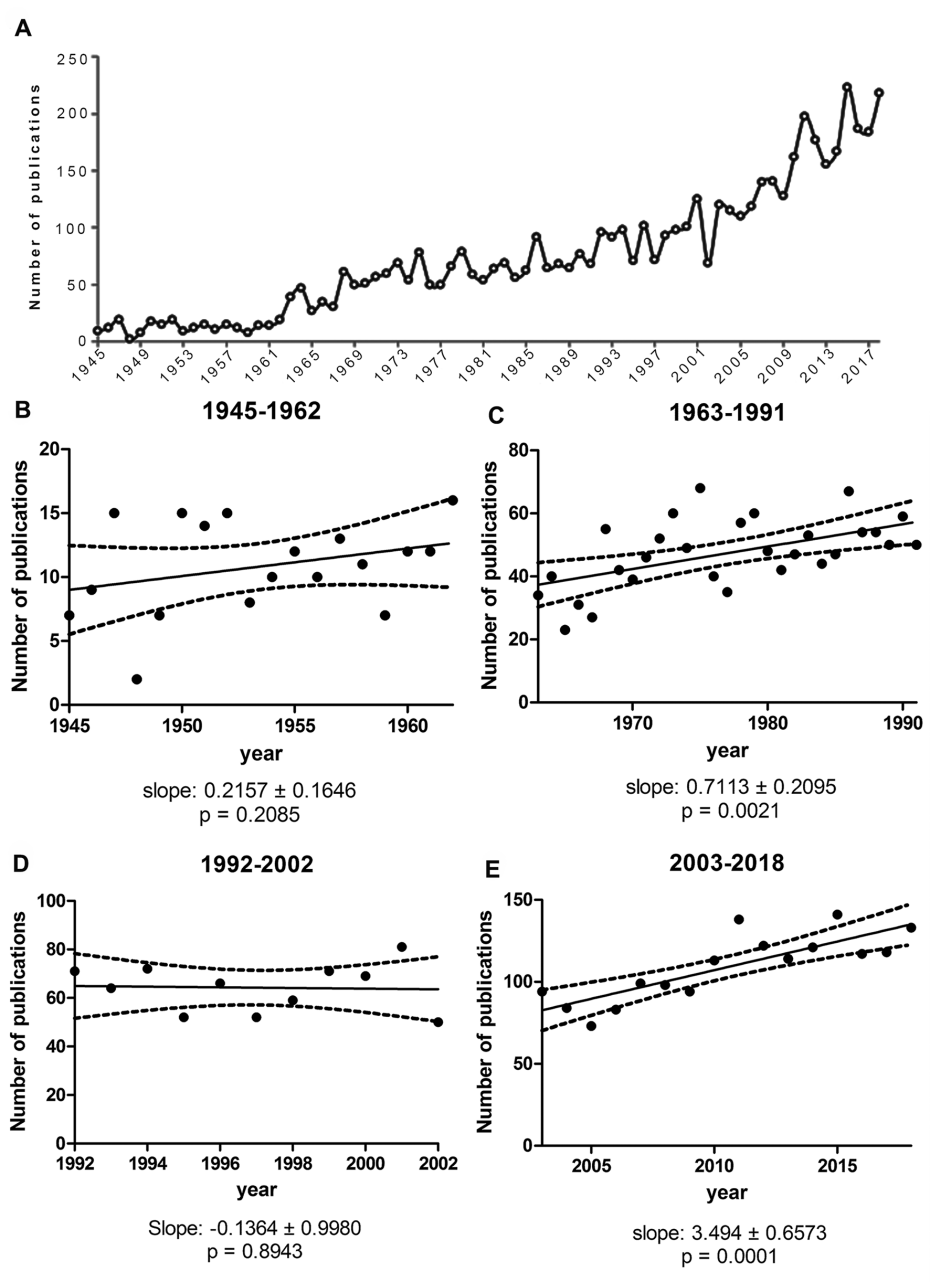
Global scientific production in the *Sporothrix* and Sporotrichosis field. ( **a**) Overall publication numbers per year (1945–2018). Trends in the number of publications on the *Sporothrix*/sporotrichosis field are represented in a linear regression form for the following time periods: ( **b**) 1945–1962; ( **c**) 1963–1991; ( **d**) 1992–2002; ( **e**) 2003–2018. The continuous line represents the best-fit line for the linear regression. Dashed lines represent the 95% confidence interval of the best-fit line.

Sporotrichosis was found to be the mycosis caused by dimorphic fungi with the lowest number of publications in both databases used in this work ( Table 1). The literature search on the *Sporothrix*/sporotrichosis field retrieved 1,868 publications from the WoS database and 3,660 publications from the Scopus database for the period of 1945 to 2018. After removing duplicate references present on both databases, new totals were 1,866 and 3,599 papers, respectively, with 1,458 publications present in both databases. The final data set resulted in 4,007 unique publications ( Figure 1; Underlying data ^23^). As depicted in Figure 2a, the overall number of publications on *Sporothrix*/sporotrichosis has increased steadily over the years. The frequency of publications reflecting the interest in the studied subject varied over time. Four periods could be identified: 1945 to 1962 ( Figure 2b), with an average of 10.8±3.7 publications per year; 1963 to 1991 ( Figure 2c), 47.2±11.3 papers per year; 1992 to 2002 ( Figure 2d), with 63.1±10.3 publications per year; and in the final period, 2003 to 2018 ( Figure 2e), 108.9±20.3 papers per year. Linear regressions of the number of publications per year were performed for each one of these periods and the results were as follows: In two of these periods, 1945–1962 and 1992–2002, the amount of publications per year was approximately constant (slopes of 0.2157 ± 0.1646 and -0.1364 ± 0.9980, *p* values of 0.2085 and 0.8943, respectively), whereas the two remaining periods, 1963–1991 and 2003–2018, presented increases in the publication numbers per year (slopes of 0.7113 ± 0.2095 and 3.494 ± 0.6573, *p* values of 0.0021 and 0.0001, respectively). The differences between the slopes were found to be extremely significant ( *p* < 0.0001).

**Table 1.** Number of publications on dimorphic fungi and their respective mycoses retrieved from Web of Science and Scopus databases (1945 – 2018).

Fungus / Disease	Number of publications per database	
	Web of Science	Scopus
*Sporothrix* / Sporotrichosis	1,868	3,660
*Paracoccidioides* / Paracoccidioidomycosis	2,747	3,692
*Histoplasma* / Histoplasmosis	7,111	11,520
*Blastomyces* / Blastomycosis	2,513	6,491
*Coccidioides* / Coccidiocomycosis	3,312	5,786

### Authorship by country

**Figure 3.**
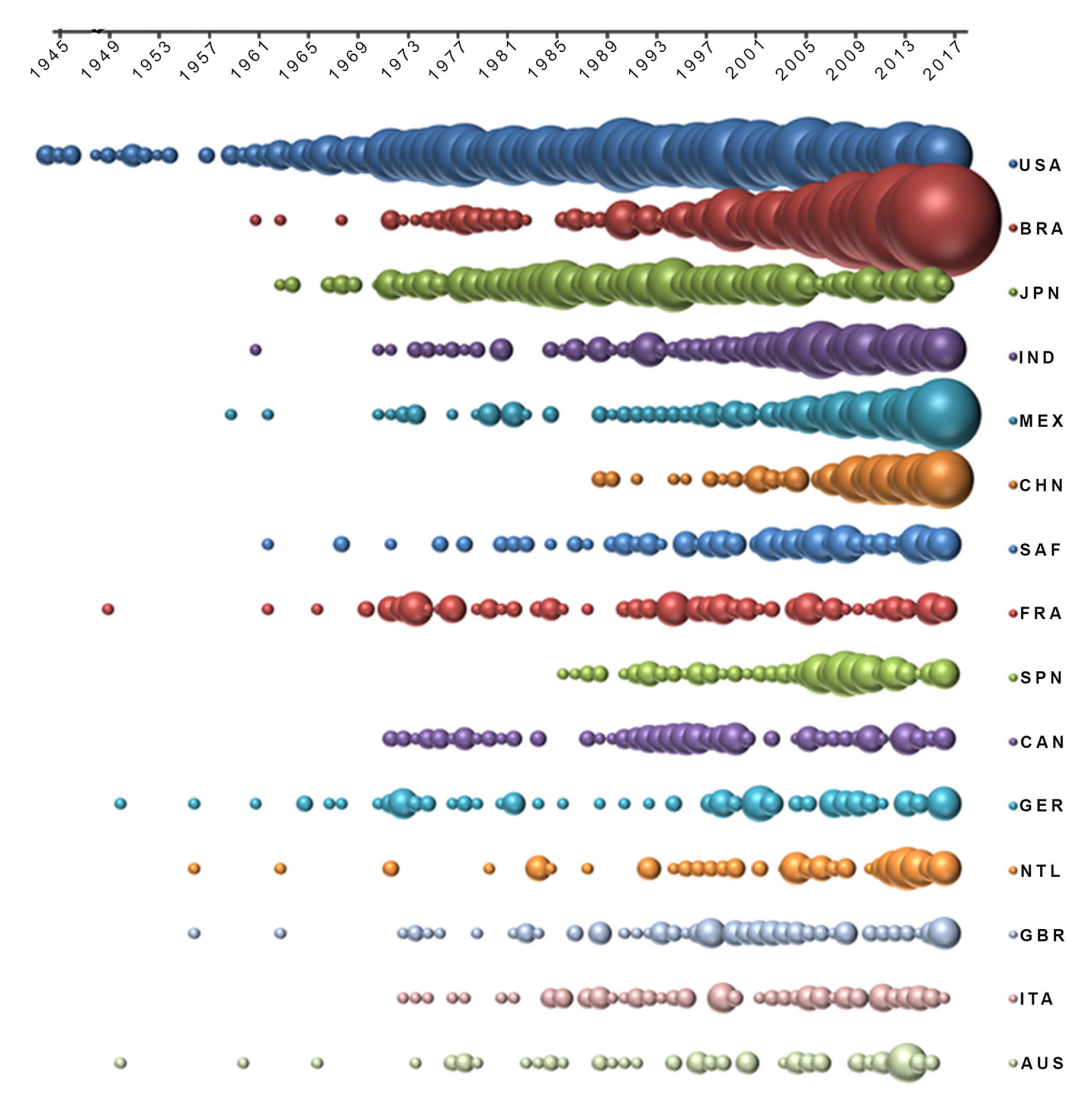
Annual publication from the top 15 countries on numbers of articles in the *Sporothrix*/sporotrichosis field. The diameter of each circle is directly proportional to the annual number of publications. USA: United States of America; BRA: Brazil; JPN: Japan; IND: India; MEX: Mexico; CHN: China; SAF: South Africa; FRA: France; SPN: Spain; CAN: Canada; GER: Germany; NTL: The Netherlands; GBR: Great Britain; ITA: Italy; AUS: Australia.

Authors from 99 countries accounted for the 4,007 publications on the *Sporothrix*/sporotrichosis field. Table 2 lists the 20 most productive countries during the entire period studied and Figure 3 depicts the annual trends of the top 15 countries. The United States of America (USA) and Brazil were the leading countries, publishing together 2,300 publications (57.4% of all publications). The frequency of publications by these two countries differed considerably. After 1973, the USA showed a regular pattern of publications with a range of 15–44 and a median of 25 papers per year, while Brazil had up to 10 publications per year until 1999, with an exponential increase in the number of publications noticed after 2000. Overall, the frequency of publications from the Japan, France, and Canada remained stable throughout the studied period, whereas a significant increase was also observed for Mexico from 2007 onwards, and China from 2009 onwards.

**Table 2.** Top 20 countries in the *Sporothrix*/sporotrichosis field (1945–2018) according to the author country of professional affiliation.

Rank	Country	Number of publications	% publications
**1**	United States of America	1,315	32.8
**2**	Brazil	985	24.6
**3**	Japan	417	10.4
**4**	India	309	7.7
**5**	Mexico	275	6.9
**6**	China	190	4.7
**7**	South Africa	155	3.9
**8**	France	137	3.4
**9**	Spain	135	3.4
**10**	Canada	130	3.2
**11**	The Netherlands	106	2.6
**12**	Germany	105	2.6
**13**	United Kingdom	96	2.4
**14**	Italy	87	2.2
**15**	Australia	74	1.8
**16**	Peru	64	1.6
**17**	Venezuela	55	1.4
**18**	Poland	49	1.2
**18**	South Korea	49	1.2
**19**	Colombia	46	1.1
**20**	Greece	44	1.1

### Scientific journals

Table 3 lists the top 15 journals (out of 1,182) with the highest number of articles on *Sporothrix*/sporotrichosis, along with their respective impact factors. Overall, the journals with most publications were Mycopathologia, Medical Mycology, Mycoses, the International Journal of Dermatology, and JAMA Dermatology. Since Brazil is the current leader on annual publications in this field ( Figure 3), the journals harboring these publications were also evaluated separately. When only publications with at least one Brazilian author were considered, in addition to Mycopathologia, Medical Mycology, Mycoses, and the International Journal of Dermatology, the journals PLoS Neglected Tropical Diseases, Frontiers in Microbiology, and four Brazilian scientific journals also appear listed among the top ten journals.

**Table 3.** Rank comparison scientific journals publishing global and Brazilian-based author articles on the *Sporothrix*/sporotrichosis field (1945–2018). The top 10 journals of each category are presented.

Journal	Country	JIF Trend 2017/18	All Publications		Brazilian Publications	
			Rank	Papers (n)	Rank	Papers (n)
Mycopathologia	Netherlands	1.476	1	283	2	68
Medical Mycology	United Kingdom	2.799	2	260	1	93
Mycoses	Germany	2.793	3	141	4	33
International Journal of Dermatology	USA	1.541	4	120	10	14
JAMA Dermatology	USA	8.107	5	96	N/A	0
Clinical Infectious Diseases	USA	9.117	6	80	13	11
Journal of Clinical Microbiology	USA	4.054	7	69	13	11
Journal of the American Academy of Dermatology	USA	6.898	8	57	21	3
Revista Iberoamericana de Micologia	Spain	0.989	9	51	14	10
Anais Brasileiros de Dermatologia	Brazil	0.884	10	50	3	37
Revista da Sociedade Brasileira de Medicina Tropical	Brazil	1.358	22	26	5	26
PLoS Neglected Tropical Diseases	USA	4.367	24	19	6	19
Frontiers in Microbiology	Switzerland	4.019	23	18	7	18
Memórias do Instituto Oswaldo Cruz	Brazil	2.833	21	16	8	16
Revista do Instituto de Medicina Tropical de São Paulo	Brazil	1.489	26	15	9	15

JIF – Journal Impact Factor N/A – Not available

### Research trends

**Figure 4.**
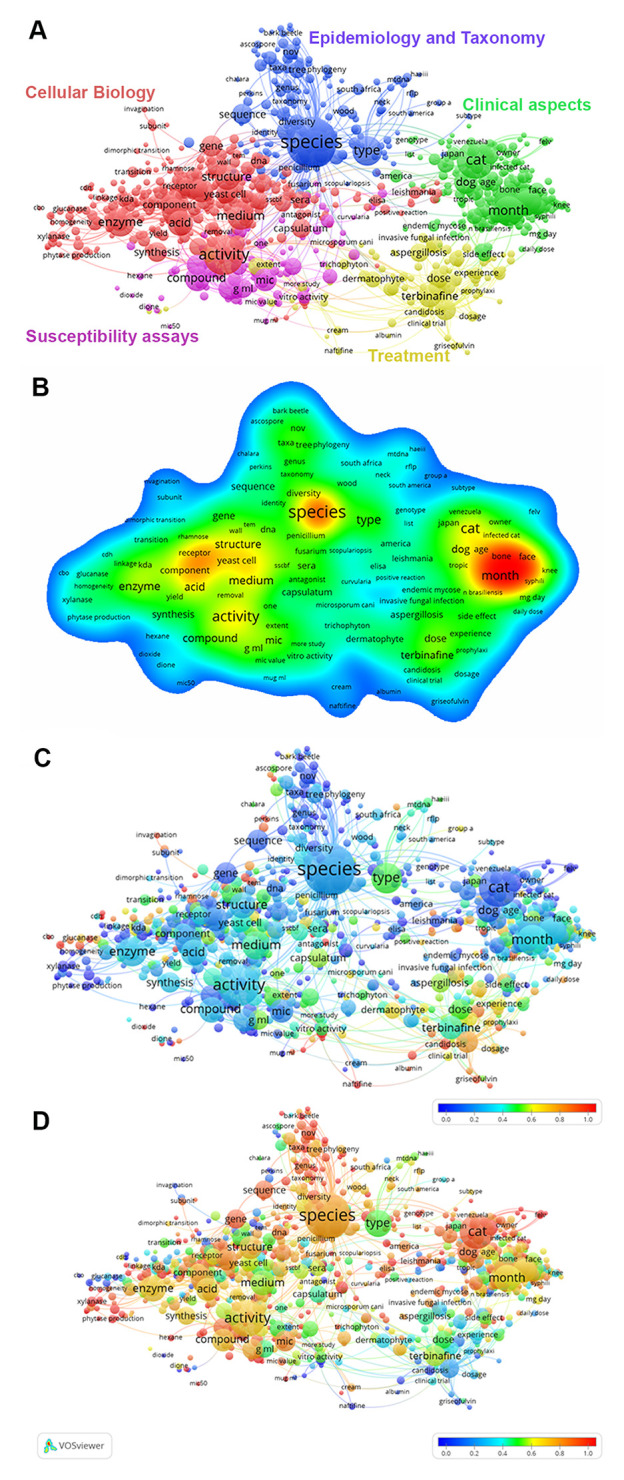
Thematic maps of *Sporothrix*/sporotrichosis research generated with the VOSviewer software. ( **a**) Terms extracted from titles and abstracts clustered into five major research areas: cellular biology, epidemiology and taxonomy, clinical aspects, treatment, and susceptibility assays. Colors indicate clusters of terms that have co-occurred more frequently in the dataset. ( **b**) The heat map shows the most frequent terms in the period analyzed (1945–2018). The frequency was graded from blue to red; where red indicates a higher frequency. ( **c**) Research trends from 1945 to 1999. ( **d**) Research trends from 2000 to 2018. The diameters of the circles on **a**, **c**, and **d** panels are directly proportional to the occurrence of each term. Lines between different circles represent relationships between terms. The colors in **c** and **d** thematic maps indicate the occurrence of a term in each period. Blue represents a low occurrence, green an average occurrence, and red a high occurrence.

The analysis of term maps revealed five broad knowledge areas of the *Sporothrix*/sporotrichosis research ( Figure 4a): description of clinical aspects (green), treatment (yellow), epidemiology and taxonomy (blue), cellular biology (red), and susceptibility assays (pink). The most frequent topics throughout the period studied are represented in a heat map format ( Figure 4b), which shows that terms about clinical aspects were more frequent, followed by terms on taxonomy and cellular biology. Since we observed that in this century publications about *Sporothrix* and sporotrichosis increased considerably ( Figure 2e), we performed an analysis in two time periods - 1945–1999 ( Figure 4c) and 2000–2018 ( Figure 4d). This assessment revealed, in this last period of highly increasing publication numbers, a shift from research focused on treatment to other knowledge areas such as epidemiology and taxonomy.

### Network of countries

**Figure 5.**
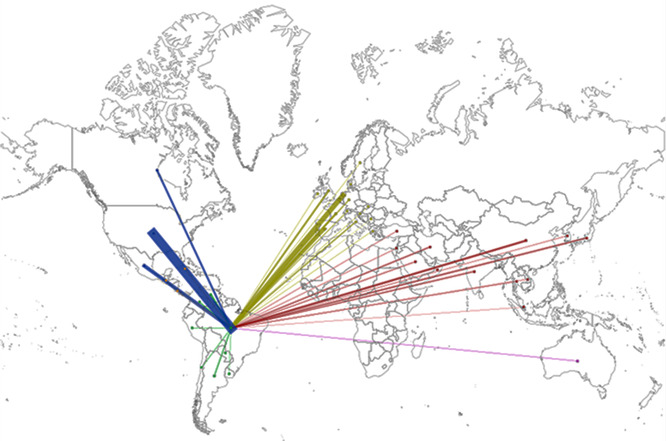
Co-authorship map between *Sporothrix*/sporotrichosis researchers. Brazilian network of scientific collaborations on the period studied. Country links were mapped based on the authors’ affiliations. Each node represents one country and two countries were considered connected if their researchers shared the authorship of a paper. Only relationships between the first author and their co-authors are shown. Links are color-coded according to the continent of the first author: North America – blue; Africa – dark green; Europe – yellow; South America – light green; Asia – red; Oceania – pink.

The international research network on *Sporothrix* and sporotrichosis was mapped according to the country affiliation of all authors. The network is formed by 99 countries, with a Brazilian leadership on collaborations. In fact, Brazilian authors have co-authorship with authors from 45 of the countries that compose the network ( Figure 5). The most frequent Brazilian collaborations were with institutions from the USA (84 publications), followed by the Netherlands (44 publications), Mexico (31 publications), Spain (23 publications), and France (16 publications).

### Network evolution among Brazilian institutions

**Figure 6.**
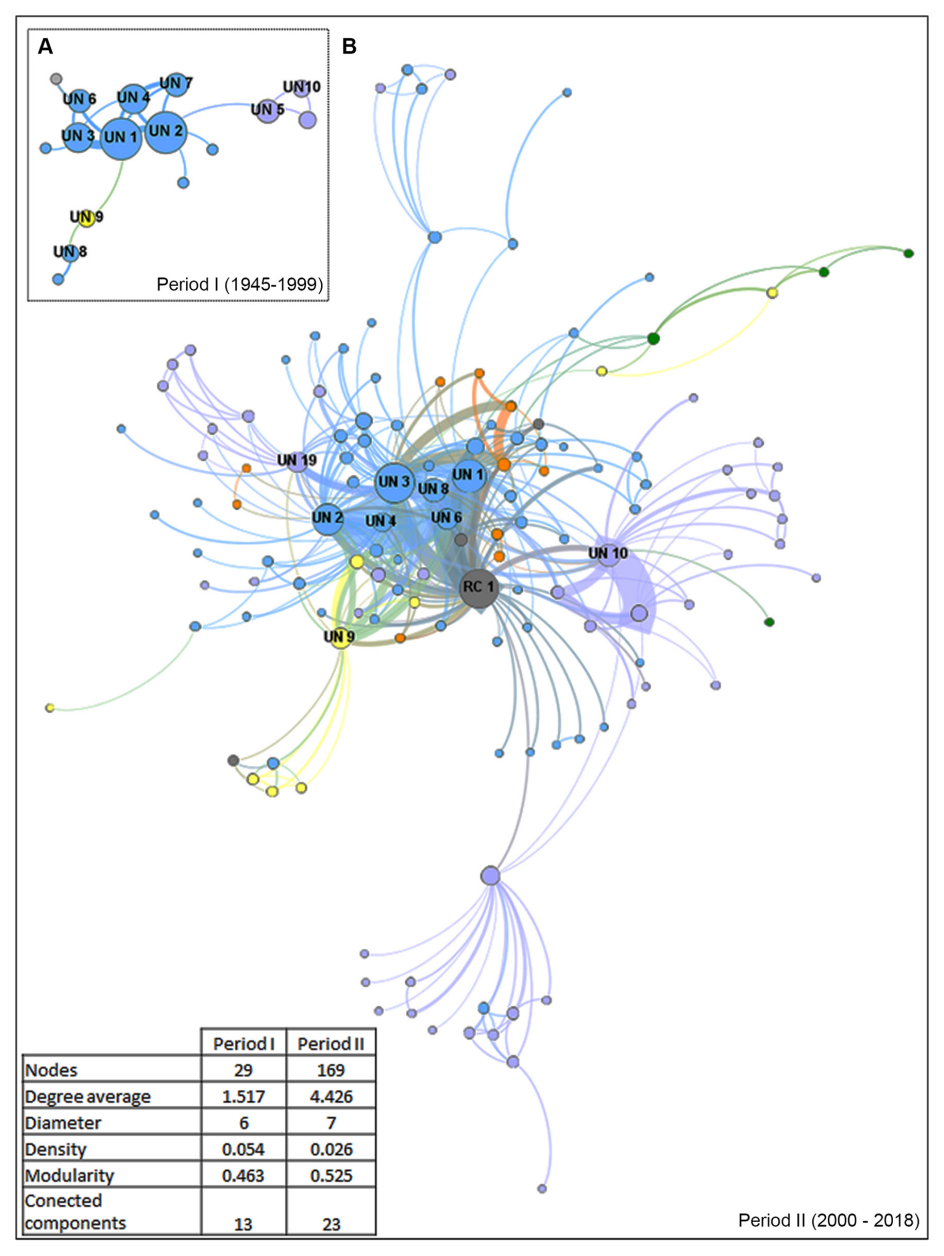
Evolution of *Sporothrix*/sporotrichosis research networks involving Brazilian institutions. ( **a**) Research network from 1945 to 1999. ( **b**) Research network from 2000 to 2018. Each node represents one institution and two institutions were considered connected if their members shared the authorship of a paper. Nodes are color coded according to the geographic location of the institutions: North region - green; Northeast region – orange; Center-West region– yellow; Southeast region- blue; and South region – lilac. Multicampus National institutions are colored in gray. The size of the nodes is proportional to their centrality degree. For visualization purposes, only the giant component is shown. The top ten Brazilian organizations with highest degree centrality are labeled in each panel.

To further understand the dynamics of the *Sporothrix*/sporotrichosis research in the current leader country of publications, the evolution of *Sporothrix* research networks in Brazil was analyzed in the two-time intervals, 1945–1999 and 2000–2018, that is, before and after the beginning of the Brazilian zoonotic endemic of sporotrichosis ( Figure 6). In total, 29 institutions, mostly from the Southeast region, were present in the first period. The network core (giant component) was formed by 16 institutions: nine universities, one public foundation, and one private laboratory, all from the Southeast region, two universities and one hospital from the South region, one university from the Midwest region and one national research center. The network degree average was 1.5, indicating low connectivity in the network ( Figure 6a). The first three institutions with higher degree centrality in that period were federal universities located in the Brazilian Southeast region ( Table 4). For the second period, the number of institutions increased almost six times. The network was composed by 169 institutions, 135 of them within the giant component. The degree average increased four times (degree average: 4.426), showing a gain in the network connectivity ( Figure 6b). A national research center appears as the most central institution in the network, followed by three federal universities, central actors in the sporotrichosis network throughout the studied period. The ten most central organizations in these networks are shown in Table 4. Notably, most of them are from the Southeast region of the country (70% and 60% for 1945–1999 and 2000–2018, respectively).

**Table 4.** Centrality Index of institutions in the Brazilian sporotrichosis collaboration network.

Rank	Period I (1945–1999)				Period II (2000–2017)			
	Institutions	Degree	Betweenness centrality	Coauthored Publications	Institutions	Degree	Betweenness centrality	Coauthored Publications
1	**UN1** **(UFRJ)**	6	58.0	38	**RC 1** **(Fiocruz)**	44	3,041.99	313
2	**UN2** **(USP)**	6	57.0	26	**UN 3** **(UNIFESP)**	43	2,092.34	133
3	**UN3** **(UNIFESP)**	4	15.0	16	**UN 1** **(UFRJ)**	37	1,810.33	115
4	**UN4** **(UNESP)**	4	7.0	9	**UN 2** **(USP)**	32	797.79	93
5	**UN5** **(UFSM)**	3	26.0	6	**UN 8** **(UFMG)**	22	851.92	34
6	**UN6** **(UERJ)**	3	14.0	10	**UN 10** **(UFRGS)**	21	1,555.58	85
7	**UN7** **(UFF)**	3	0.0	4	**UN 9** **(UNB)**	18	694.66	27
8	**UN8** **(UFMG)**	2	14.0	10	**UN 19** **(UFPR)**	18	650.58	21
9	**UN9** **(UNB)**	2	26.0	2	**UN 6** **(UERJ)**	18	394.06	105
10	**UN10** **(UFRGS)**	2	0.0	2	**UN 4** **(UNESP)**	16	766.62	75

UFRJ: Universidade Federal do Rio de Janeiro, Southeast Brazil; USP: Universidade de São Paulo, Southeast Brazil; UNIFESP: Universidade Federal de São Paulo, Southeast Brazil; UNESP: Universidade Estadual Paulista, Southeast Brazil; UFSM: Universidade Federal de Santa Maria, South Brazil; UERJ: Universidade do Estado do Rio de Janeiro, Southeast Brazil; UFF: Universidade Federal Fluminense, Southeast Brazil; UFMG: Universidade Federal de Minas Gerais, Southeast Brazil; UNB: Universidade de Brasília, Center Western Brazil; UFRGS: Universidade Federal do Rio Grande do Sul, South Brazil; UFPR: Universidade Federal do Paraná, South Brazil; Fiocruz: Fundação Oswaldo Cruz, Brazil.

### Research funding

The analysis of funding acknowledgments was used as proxy information for research funding. A total of 457 (34%) articles out of 1,310 publications acknowledged funding during the period 2012 to 2018. Table 5 shows the top 10 funding agencies and the number of publications out of a total 236 funding organizations and initiatives. Five of them are from Brazil, two from Mexico, one from China, one from the USA, and one from South Africa.

**Table 5.** Top 10 funding organizations supporting publications on *Sporothrix*/sporotrichosis (2012 – 2018).

Rank	Major Funding Organization	Number of funded publications (%)	Country
1	CNPq - Conselho Nacional de Desenvolvimento Científico e Tecnológico	145 (11)	Brazil
2	CAPES - Coordenação de Aperfeiçoamento de Pessoal de Nível Superior	84 (6.4)	Brazil
3	FAPERJ - Fundação Carlos Chagas de Amparo à Pesquisa do Estado do Rio de Janeiro	64 (4.8)	Brazil
4	FAPESP - Fundação de Amparo à Pesquisa do Estado de São Paulo	61 (4.6)	Brazil
5	National Natural Science Foundation of China	36 (2.7)	China
6	UG - Universidad de Guanajuato	34 (2.6)	Mexico
7	CONACyT - Consejo Nacional de Ciencia y Tecnología	33 (2.5)	Mexico
8	FIOCRUZ - Fundação Oswaldo Cruz	32 (2.4)	Brazil
9	NIH - National Institutes of Health	22 (1.6)	United States of America
10	DST/NRF Centre of Excellence in Tree Health Biotechnology (CTHB)	15 (1.1)	South Africa

### Statements about practice

**Figure 7.**
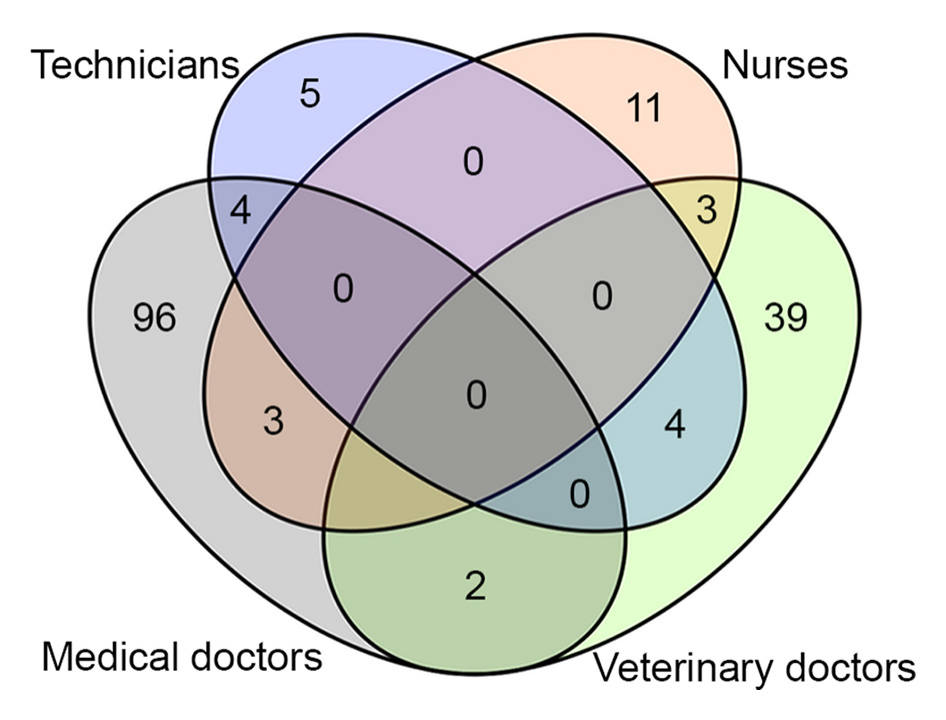
Identification of specified audiences in publications about *Sporothrix* or sporotrichosis. The Venn diagram was constructed based on four different audiences observed in the abstracts of the publications studied. Numbers on diagrams indicate how many abstracts reporting advice for medical doctors, veterinary doctors, nurses, and/or lab technicians were identified.

An exploratory study on publications was conducted to assess the potential methodology for retrieve actionable messages. After excluding 1,262 articles with no published abstract, the type of language and the intended audience of the publications were assessed for 2,745 articles ( Table 6). Since Brazil was the current leading country publishing in the *Sporothrix*/sporotrichosis field, we compared the frequency of statements related to research and practice on papers with at least one Brazilian author with those of authors from other countries exclusively. The proportion of papers presenting language compatible with tentative ( *p* = ** 0.0003) or minimal advice ( *p* = 0.0139) statements for sporotrichosis research was higher in the papers co-authored by Brazilians. The specific audience for the statements was mentioned in 167 abstracts. As depicted in Figure 7, most of the implications for practice were directed to medical doctors, followed by veterinarians, nurses, and laboratory technicians. Only 16 publications presented more than one audience, as indicated at the abstract. Some examples of actionable messages and their audiences are listed in Table 7.

**Table 6.** Type of language statements and identified audience from *Sporothrix* and sporotrichosis articles (1945–2018).

Statements with	Brazil	Other countries	p-value
Prescriptive language	52 (9.5%)	226 (10.3%)	0.6576
Tentative language	195 (35.7%)	613 (27.9%)	0.0003
Minimal advice	78 (14.3%)	230 (10.4%)	0.0139
Audience specified	37 (6.8%)	122 (5.5%)	0.2714

**Table 7.** Examples of actionable messages found in article abstracts from papers evaluated in this study.

Title of publication	PMID	Type of language	Audience Specified	actionable message
Histopathology of canine sporotrichosis: A morphological study of 86 cases from Rio de Janeiro	19360480	Prescriptive	No	Specific staining of serial sections is recommended in the case of dogs with skin lesions whose histopathological presentation is consistent with sporotrichosis.
Sporotrichosis in cats: ABCD guidelines on prevention and management	23813827	Prescriptive	Yes	Professionals must wear gloves when handling cats with skin nodules and ulcers and dealing with diagnostic samples
Molecular cloning, characterization and differential expression of DRK1 in *Sporothrix schenckii*	23175272	Tentative	No	SsDRK1 may be involved in the dimorphic switch in *S. schenckii*.
Disseminated sporotrichosis with extensive cutaneous involvement in a patient with AIDS	10025867	Tentative	Yes	It is important that clinicians be aware of the presentation of this unusual opportunistic infection
Sporotrichosis in Himachal Pradesh (north India)	10492787	Minimal advice	No	This study identifies Kangra district and adjoining areas in Himachal Pradesh as an endemic region for sporotrichosis and highlights the need for evaluation of geo-climatically similar areas.
Unsuspected sporotrichosis in childhood	11332673	Minimal advice	Yes	We urge clinicians to consider sporotrichosis in the differential diagnosis of a solitary skin nodule.

## Discussion

The results herein presented demonstrate that research on *Sporothrix* and sporotrichosis ** is increasing worldwide. This increase could be even greater, because some regional or specialized journals may possibly not be indexed in the databases reviewed. However, considering the proposed focus, the authors believe that the study material was a comprehensive representation of the scientific production in the field.

 The apparent fast engagement of authors from several countries in the late 90s can be explained by the global increase in the incidence of sporotrichosis ^1^, especially in Brazil ^2^ and China ^24^, two of the major countries currently publishing on this field. Historically, Brazil and China are also the leading countries reporting sporotrichosis cases, followed by South Africa, all of them with more than 3,000 human and animal cases reported ^25^. In this study, South Africa ranked as the seventh country on number of publications with no increasing trend. Most of the cases in this country occurred during an outbreak in the 1940’s ^26^. Although a new mine-related outbreak occurred more recently ^27^, there is no evidence that there is a re-emergence of sporotrichosis occurring in South Africa, which may explain its apparently constant number of publications in this field.

The number of publications on the *Sporothrix*/sporotrichosis ** field usually followed crucial events that have driven to a shift on typical clinical cases of sporotrichosis. In the 1980s, with the development of ketoconazole, the first oral antifungal azole drug ^28^, and the emergence of AIDS, there was an increase in papers reporting results of sporotrichosis treatment ^29, 30^ and unusual severe forms of sporotrichosis related to immunosuppression ^31– 33^, coherent with the predominant thematic at the time. After 2007, the advances in polyphasic taxonomy of *Sporothrix* spp., driven by the worldwide increasing numbers of clinical cases ^5^, resulted in the description of several new pathogenic *Sporothrix* species ^5– 7^, which may explain the shift for epidemiology and taxonomy occurred in this century.

Authors from Brazil and the USA authored around 57% of the papers on the subject studied. Also, a high-level of collaboration between these two countries was seen, as occurred in other knowledge areas ^34, 35^. While the number of publications authored by researchers from the USA showed a consistent trend in the studied period, Brazilian authors have emerged as very productive in this field. Some factors that may have influenced the strong commitment of Brazilian researchers in the *Sporothrix*/sporotrichosis field include: (i) the zoonotic sporotrichosis epidemic, that begun in Rio de Janeiro state in 2000 ^36^ and now is spreading to almost the totality of the Brazilian territory ^37– 39^; (ii) the increasing numbers of national and international Brazilian collaboration networks ^40^; and (iii) the beginning and still discrete recognition of the relevance of this research field by Brazilian funding agencies, which was detected in the funding analysis of this study.

The evaluation of co-authorship networks in sporotrichosis identified structural patterns of research involving Brazilian scientists. USA, Netherlands, Mexico, Spain, and France were Brazil’s most frequent collaborators. Government collaboration and research programs supported by the USA are in place since 1950’s, explaining the robust scientific collaboration between the countries ^34^.

Our initial analysis of the four periods revealed that two of them, 1945-1962 and 1992-2002, publications remained without significant variation. The strongest variation occurred after 2002, which coincides with the start of publications about the Brazilian zoonotic epidemics of sporotrichosis. Therefore, for subsequent analysis, we chose to separate the articles only in two periods: before and after the beginning of the Brazilian sporotrichosis epidemics. The Brazilian sporotrichosis research network has grown, almost six times in size, from the first (initial 54 years of low productivity) to the second period (last 18 years with a high level of publications), over the seven decades evaluated. This fact, together with the increase in the average degree and size of the giant component, that is, the network core, indicated a strengthening of network cohesion for collaboration over the years. It is worth noting that the measure of centrality is not related to the volume of publications, but to the capacity to aggregate collaboration. The centrality analysis of the interinstitutional network highlighted the current role of one national research center in promoting collaboration and knowledge spreading in the sporotrichosis field. This same research center also has a centrality on the research of other infectious diseases ^15, 41^. High degree centrality from a research center indicated a strong collaborative pattern in research ^15^. Together with other institutions, the Federal Universities in Southeast Brazil had a vital role in maintaining the connection between the overall research network and in ensuring that the less well-connected organizations gained access to new knowledge and information on sporotrichosis during the period studied. It is interesting to note that just recently, sporotrichosis has spread to the Northeast ^39^ and Midwest regions of the country ^38^, which means that, in the future, institutions from these regions may have a more important role in the collaboration network.

The network evolution was accompanied by a shift in the research trends. When comparing the first period to the 2000 onwards, the basic biomedical research profile gained more importance and became most frequent. The diversity of research trends may be related to the continued increase of institutions engagement in the *Sporothrix*/sporotrichosis network, providing new insights through new collaborations, showing the effectiveness of the research network in knowledge generation, sharing and diffusion. A similar scenario was observed in dengue research networks ^42^.

The current funding scenario for fungal infections has a negative perspective. Only cryptococcal meningitis was classified within the most poorly funded neglected diseases by the G-Finder survey (a reference publication for research funding flows on neglected infectious), receiving less than 0.5% of global funding ^43^. Other clinically relevant fungal infections (paracoccidioidomycosis, mycetoma, sporotrichosis, and chromoblastomycosis) were not even quoted in the G-Finder report. In fact, a recent study noticed that some fungal diseases, including sporotrichosis, have received negligible funding ^44^. Our study corroborates these findings, showing that, with the exception of Brazil, possibly because of the expanding sporotrichosis endemic areas ^37– 39^, other countries are not strongly committed to sporotrichosis funding research.

This work aimed to evaluate the applicability of a methodology proposed to retrieve key messages from abstracts. Traditionally, the scientific publication is the most frequent method of disseminating research results for a possible transfer of knowledge. In the ideal scenario, scientific articles and their abstracts should present a summary of the results, as well as the main conclusions and recommendations, when appropriate. According to the World Health Organization (WHO), five aspects should be taken into account for knowledge transfer: i) the presence of a clear message; ii) defined target audience; iii) communication strategy; iv) the method of transfer and, finally; v) the evaluation of the effect of the transferred message. Identifying the first two of these five points in a publication is key to bringing the results to action ^45^. In our analysis, we recovered in publications co-authored by Brazilian researchers more words related to tentative and minimal advice than in papers written by authors from other countries. This could be related to a concern of Brazilian researchers to address the major public health problem that sporotrichosis has become in the country ^2, 37^, which has certainly affected the overall number of publications. The screening method proved to be an efficient tool to identify and recovery recommendation messages in the abstracts. New words should be added to the recovery method to improve its sensitivity (For example: need, ought, necessary, reinforce, advice, highlight).

The fact that some journals have a specific audience (for instance, four of the top ten journals are directed to medical doctors interested in dermatology) may bias the nature of recommendations. However, as abstracts are the first contact for the readers with a publication, we believe mentioning statements or recommendations for practice in this section can improve the visibility of the research. Also, the analysis of contents revealed that a minor proportion of publications is directed to more than one specific audience, which is one indicator of a poor translational research in the studied field.

This study is based on a bibliometric approach to observe the evolution of the world scientific literature and identify research trends of sporotrichosis field, focusing in the Brazilian scenario. As a methodological approach, some national, regional or specialized journals indexed in regional databases such as SciELO and LILACS were not included, due to the difficulty to obtain total equivalence for a global analysis, since other regional databases should be included as well, making data harmonization difficult.

The retrieving key messages wording review based solely on abstracts is another limitation of this study. However, abstracts are the most widely read summaries of research findings and are an important source of information for clinicians and policy makers; particularly abstracts published in high profile journals. In this work, the proposal was validate the methodology of recovering. Future analysis is necessary to understand the translation scientific research into policies and practical applications.

Despite cases being reported all around the globe ^1^, sporotrichosis remains a neglected disease in terms of research interest, funding and medical attention. The growth of research on sporotrichosis needs to be translated into practical applications on diagnosis, treatment, and prevention, given the limited tools available for rapid tests ^2^, the cost-effective treatment ^46^, and the lack of effective vaccines ^47^. The challenge is to share and advance knowledge to curb this disease. The funding agencies have a critical role to play in this context.

## Data availability

Underlying data generated in this study are available at Open Science Framework: Bibliometric assessment of sporotrichosis research. https://doi.org/10.17605/OSF.IO/MXU6V ^23^

This project contains the following underlying data:

- (F1_T1) Countries publication by years.xlsx (All countries with sporotrichosis publications)
- (F2) MapVoS.txt (Map file for VOSViewer software)
- (F2) NetVoS.txt (Network file for VOSViewer software)
- (F3) CountryNet.gephi (Country map file for Gephi software)
- (F4) Period I.gephi (Network file for Gephi Software)
- (F4) Period II.gephi (Network file for Gephi Software)
- (T2) Source.xlsx (All Journals with retrieved publications about sporotrichosis)
- (T3) Funding BR 12_18.xlsx (Funding agencies supporting sporotrichosis research)
- (T4) Actionable messages.xlsx (implications for practice on retrieved articles)

Data are available under the terms of the Creative Commons Zero "No rights reserved" data waiver (CC0 1.0 Public domain dedication).
