# Supplementary material for: Bibliometric assessment and key messages of sporotrichosis research (1945-2018)
Source: F1000Res. 2021 Apr 9;9:654. Originally published 2020 Jun 29. [Version 2] doi: 10.12688/f1000research.24250.2 (PMC8080985; doi:10.12688/f1000research.24250.2)
Supplement: Peer review history

# Peer review: Reviewer response for version 2

**DOI:** 10.5256/f1000research.55785.r83080

**Published:** 2021-05-25

**Authors:** Maria Simone Menezes Alencar

**Affiliations:** 1 Federal University of the State of Rio de Janeiro, Rio de Janeiro, Brazil

The review greatly improved the work. No further comments.

Is the work clearly and accurately presented and does it cite the current literature?

Yes

If applicable, is the statistical analysis and its interpretation appropriate?

Yes

Are all the source data underlying the results available to ensure full reproducibility?

Yes

Is the study design appropriate and is the work technically sound?

Partly

Are the conclusions drawn adequately supported by the results?

Yes

Are sufficient details of methods and analysis provided to allow replication by others?

Yes

Reviewer Expertise:

Scientific and technological information acting mainly on the following subjects: bibliometrics, technological foresight, patent analysis and open science.

I confirm that I have read this submission and believe that I have an appropriate level of expertise to confirm that it is of an acceptable scientific standard.

# Peer review: Reviewer response for version 2

**DOI:** 10.5256/f1000research.55785.r83081

**Published:** 2021-04-23

**Authors:** Max Carlos Ramírez-Soto

**Affiliations:** 1 School of Public Health and Administration, Universidad Peruana Cayetano Heredia, Lima, Peru

I have no comments. The authors have raised the observations.

Is the work clearly and accurately presented and does it cite the current literature?

Yes

If applicable, is the statistical analysis and its interpretation appropriate?

Yes

Are all the source data underlying the results available to ensure full reproducibility?

No

Is the study design appropriate and is the work technically sound?

Partly

Are the conclusions drawn adequately supported by the results?

Partly

Are sufficient details of methods and analysis provided to allow replication by others?

Yes

Reviewer Expertise:

Medical Mycology

I confirm that I have read this submission and believe that I have an appropriate level of expertise to confirm that it is of an acceptable scientific standard.

# Peer review: Reviewer response for version 1

**DOI:** 10.5256/f1000research.26752.r77886

**Published:** 2021-02-24

**Authors:** Daniel Zamith-Miranda

**Affiliations:** 1 Division of Infectious Diseases, Department of Medicine, Albert Einstein College of Medicine, New York, NY, USA

The manuscript "Bibliometric assessment and implications for practice of sporotrichosis research (1945-2018)" from Albuquerque and colleagues shows how research on sporotrichosis has been conducted in the mentioned time range. The authors raised relevant data to conclude where and how this research has been done. Besides usual typos, parts of the text, as well as some figures should be remade (comments below).

**Introduction**

 When citing reference #15, the authors should highlight that the cited bibliometric analyses were about tuberculosis. Otherwise, the reader may think that those analyses were already made for sporotrichosis and that would decrease the impact of the current work.

 End of third paragraph: “impact” is more appropriate than “impacts”.

**Methods**

 Is citation #19 correctly referenced? Isn’t the publishing year supposed to be included at least?

**Results**

 What is reference 23? Is it a preprint? Is it the same work as this manuscript published somewhere else?

 Table 3: Why does the Rank of all publications, after going from 1 – 10, go to 22, 24, 23, 21, and 26? Isn’t that supposed to be the top 15 journals?

 Figure 4: From A to D, most of what is written is way too small. Even when zooming in, some words are difficult to read. I recommend the authors to at least increase the resolution, but also try to rearrange the panels in order to make it easier for the reader. Even 4c and 4D have distinct resolution, even though the text is supposed to be identical among them.

 Fig. 4 Legend: “( **b**) The heat map shows the most frequent terms in the period analyzed”. Please specify the period.

 Fig 5A: I am not sure if this figure is adding information or even helping the understanding of the text. The authors should consider to change the format, or even take it out of the manuscript.

 Fig 6: It seems that, besides the research networks, this figure is also about the distribution of scientific institutions in Brazil. Merging the current figure with a map would make it easier to visualize.

 Table 7: Consider to keep it as supplementary information.

**Discussion**

 In “…This fact, together with the increase in the average degree and size of the giant component…”. Please clarify what is the giant component.

 In “…It is interesting to note that just recently, sporotrichosis has spread to the Northeast and Center-Western regions of the country…”. For the sake of being consistent with the nomenclature used in Results, please change Center-Western to Midwest.

 In “…In our analysis, publications co-authored by Brazilians researchers have more tentative and minimal…”, change to Brazilian researchers.

 I agree with the other reviewer that conclusions regarding implications in practice cannot be taken based on the method used.

Is the work clearly and accurately presented and does it cite the current literature?

Yes

If applicable, is the statistical analysis and its interpretation appropriate?

Yes

Are all the source data underlying the results available to ensure full reproducibility?

Partly

Is the study design appropriate and is the work technically sound?

Yes

Are the conclusions drawn adequately supported by the results?

Partly

Are sufficient details of methods and analysis provided to allow replication by others?

Yes

Reviewer Expertise:

Microbiology (mycology) and immunology.

I confirm that I have read this submission and believe that I have an appropriate level of expertise to confirm that it is of an acceptable scientific standard, however I have significant reservations, as outlined above.

# Response

**Published:** 2021-03-29

**Authors:** Rodrigo Almeida-Paes

**Affiliations:** Fundacao Oswaldo Cruz, Brazil

The authors thank the careful revision of our manuscript. Our point-by-point response to the reviewers comments are presented below:

**Reviewer comment: **When citing reference #15, the authors should highlight that the cited bibliometric analyses were about tuberculosis. Otherwise, the reader may think that those analyses were already made for sporotrichosis and that would decrease the impact of the current work. End of third paragraph: “impact” is more appropriate than “impacts”.

**Our response and action taken: **Thank you for the corrections. We have added reference #15 to emphasize the potential of network analysis. We have clarified the article findings as suggested.

**Reviewer comment: **Methods: Is citation #19 correctly referenced? Isn’t the publishing year supposed to be included at least?

**Our response and action taken: **Thank you for the corrections. All references were carefully reviewed and corrected as suggested. The correct year is 2009.

**Reviewer comment: **Results: What is reference 23? Is it a preprint? Is it the same work as this manuscript published somewhere else?

**Our response and action taken: **This reference refers to the raw data generated in this study that are available at Open Science Framework: Bibliometric assessment of sporotrichosis research. https://doi.org/10.17605/OSF.IO/MXU6V This was a requirement of the journal.

**Reviewer comment: **Table 3: Why does the Rank of all publications, after going from 1 – 10, go to 22, 24, 23, 21, and 26? Isn’t that supposed to be the top 15 journals?

**Our response: **Our intention with this table is to show the top 10 global journals and the top 10 journals publishing Brazilian research. Sometimes a journal within the Brazilian top 10 is not within the top 10 global journals, PLoS Neglected Tropical Diseases, for instance (top 6 Brazilian and 24th global).

**Action taken: **Table 3 caption was amended to include the description of rank comparison.

**Reviewer comment: **Figure 4: From A to D, most of what is written is way too small. Even when zooming in, some words are difficult to read. I recommend the authors to at least increase the resolution, but also try to rearrange the panels in order to make it easier for the reader. Even 4c and 4D have distinct resolution, even though the text is supposed to be identical among them.

 Fig. 4 Legend: “(b) The heat map shows the most frequent terms in the period analyzed”. Please specify the period.

**Our response and action taken: **Regarding Figure 4 resolution, we gave our best to increase font letters as requested but, to do it, figure would be larger than the page size. However, all terms that generated the figure are presented in the (F2) NetVoS.txt (Network file for VOSViewer software) underlying data, available at https://doi.org/10.17605/OSF.IO/MXU6V. The legend of the figure was rewritten, to comply with the reviewer's request.

**Reviewer comment:** Fig 5A: I am not sure if this figure is adding information or even helping the understanding of the text. The authors should consider to change the format, or even take it out of the manuscript.

**Our response and action taken: **According to the reviewer suggestion, the figure was removed from the manuscript.

**Reviewer comment: **Fig 6: It seems that, besides the research networks, this figure is also about the distribution of scientific institutions in Brazil. Merging the current figure with a map would make it easier to visualize.

**Our response: **Unfortunately, we were not able to merge this figure with a map. We apologize for this situation.

**Reviewer comment: **Table 7: Consider to keep it as supplementary information.

**Our response: **F1000Research does not accept supplementary material, so we kept the table within the main article.

**Reviewer comment: **In “…This fact, together with the increase in the average degree and size of the giant component…”. Please clarify what is the giant component.

**Our response and action taken: **The giant component is the network core (Mentioned in the results section - Network evolution among Brazilian institutions). This information was added to the text.

**Reviewer comment: **In “…It is interesting to note that just recently, sporotrichosis has spread to the Northeast and Center-Western regions of the country…”. For the sake of being consistent with the nomenclature used in Results, please change Center-Western to Midwest.

**Our response and action taken: **The text was changed accordingly.

**Reviewer comment: **In “…In our analysis, publications co-authored by Brazilians researchers have more tentative and minimal…”, change to Brazilian researchers.

**Our response and action taken: **The text was changed accordingly.

**Reviewer comment: **I agree with the other reviewer that conclusions regarding implications in practice cannot be taken based on the method used.

**Our response and action taken: **As requested by all reviewers, ****the text has been rewritten to demonstrate the proposal for key message recovery methodology and not to conclude implications for practice.

# Peer review: Reviewer response for version 1

**DOI:** 10.5256/f1000research.26752.r77889

**Published:** 2021-02-15

**Authors:** Maria Simone Menezes Alencar

**Affiliations:** 1 Federal University of the State of Rio de Janeiro, Rio de Janeiro, Brazil

The topic of the work is relevant, and the bibliometric approach is adequate. The content analysis proposal is innovative and deserves to be highlighted.

 It is strongly recommended that searches in bibliometric studies be conducted in specialized databases. Although the used methodology which includes data from several sources is strong, the study should include other data sources in the field. I also suggest the inclusion of the Medline database in this study.

 I suggest that only articles published in journals be used, as they are more relevant documents that report completed research. Peer-reviewed journals provide scientific credibility and are not as biased as other indexed documents, such as letters, notes, editorials, etc.

 Although the methodological stage “Implications for practice” shows an interesting innovation in its approach, it should be interpreted with care. To validate the results, I suggest that this stage be improved with two independent experts analyzing a statistical percentage of the sample.

 Although the analysis of studies on the subject in the four periods shows different behaviors, these ranges are not used in other analyses. In this way, this presentation is somewhat disconnected with the general context of the article.

 The other results are well presented and consistently discussed.

Is the work clearly and accurately presented and does it cite the current literature?

Yes

If applicable, is the statistical analysis and its interpretation appropriate?

Yes

Are all the source data underlying the results available to ensure full reproducibility?

Yes

Is the study design appropriate and is the work technically sound?

Partly

Are the conclusions drawn adequately supported by the results?

Yes

Are sufficient details of methods and analysis provided to allow replication by others?

Yes

Reviewer Expertise:

Scientific and technological information acting mainly on the following subjects: bibliometrics, technological foresight, patent analysis and open science.

I confirm that I have read this submission and believe that I have an appropriate level of expertise to confirm that it is of an acceptable scientific standard, however I have significant reservations, as outlined above.

# Response

**Published:** 2021-03-29

**Authors:** Rodrigo Almeida-Paes

**Affiliations:** Fundacao Oswaldo Cruz, Brazil

We would like to thank this reviewer for her helpful comments that helped to improve the quality of our manuscript.

 Our point-by-point responses are presented below:

**Reviewer comment: **It is strongly recommended that searches in bibliometric studies be conducted in specialized databases. Although the used methodology which includes data from several sources is strong, the study should include other data sources in the field. I also suggest the inclusion of the Medline database in this study. I suggest that only articles published in journals be used, as they are more relevant documents that report completed research. Peer-reviewed journals provide scientific credibility and are not as biased as other indexed documents, such as letters, notes, editorials, etc.

**Our response: **The authors chose as methodological approach not to use regional bases as  SciELO and LILACS, due to the difficulty to obtain total equivalence for a global analysis, since other regional bases should be included and the difficulty of  data harmonization. Scopus includes a more expanded spectrum of journals (covers 100% of Medline and Embase) and Web of Science covers the oldest publications. Also, metadata for multiple affiliations for each author or contributor are included in Pubmed only at the end of 2014.

**Action taken: **The sentence in methods was altered in order to clarify the point raised by the reviewer identifying the different types of articles analyzed (original articles, reviews, letters and editorials). Please see Souce of data section.

**Reviewer comment: **Although the methodological stage “Implications for practice” shows an interesting innovation in its approach, it should be interpreted with care. To validate the results, I suggest that this stage be improved with two independent experts analyzing a statistical percentage of the sample.

**Our response: **We appreciate the sugestion. The proposal of this research was to evaluate a methodology to identify messages and technical recommendations for action in the abstracts of scientific publications on sporotrichosis. In this first moment, the authors do not intend to analyze the messages, but the validation of the recovery methodology. And, in fact, two of the authors reviewed the data. In the further analysis, in order to validate the recovery of recommendations, two independently experts will always review the recommendations.

**Action taken: **The text has been modified to clarify that this was a methodological evaluation proposal, rather than a comprehensive analysis of implications for practice. We also clarified that two evaluators collected the data.

**Reviewer comment: **Although the analysis of studies on the subject in the four periods shows different behaviors, these ranges are not used in other analyses. In this way, this presentation is somewhat disconnected with the general context of the article. The other results are well presented and consistently discussed.

**Our response: **The reviewer raised an important point. Our initial analysis of the four periods revealed that two of them, 1945-1962 and 1992-2002, publications remained without significant variation. The strongest variation occurred after 2002, which coincides with the start of publications about the Brazilian zoonotic epidemics of sporotrichosis. Therefore, for subsequent analysis, we chose to separate the articles only in two periods: before and after the beginning of the Brazilian sporotrichosis epidemics.

**Action taken:** The issue raised by the reviewer was clarified in the research trends section of results and further addressed in the discussion.

****

# Peer review: Reviewer response for version 1

**DOI:** 10.5256/f1000research.26752.r71160

**Published:** 2020-09-24

**Authors:** Max Carlos Ramírez-Soto

**Affiliations:** 1 School of Public Health and Administration, Universidad Peruana Cayetano Heredia, Lima, Peru

Congratulations to the authors. Despite sporotrichosis remains a neglected disease in terms of research interest, funding and medical attention, to which the authors have made significant contributions. However, this study has several major observations:

**Methods.**

 The literature search is limited to two databases. Fig. 1. Present a flow, and follow the guidelines of a scoping review.

 MedLine / Pubmed and Abstracts of Scientific Conference should be reviewed.

 There are several published studies on sporotrichosis in journals indexed in SCIELO, LiLACS, etc. These papers have not been included in the study.

 I don’t think that this article contains enough robust data to evidence the implications for practice. The strategy and organization of the information about Implications for practice is not appropriate. A set of words related to tentative language is biased. Furthermore, only the abstracts have been reviewed. To evaluate the implications in practice, it is necessary to organize the different types of published studies: case series, observational study, clinical trials and systematic review, diagnostic test, etc. Furthermore, studies not related to the clinical practice of sporotrichosis should be excluded.

**Results:**

 Paragraph 1. I suggest include in Results section include the statistical test.

 Scientific production by hyperendemic areas of sporotrichosis should be presented.

 Table 3. I suggest to make a comparison between Brazil, USA, Mexico, China, or hyperendymic areas of sporotrichosis.

 The authors should include the different types of published articles: review, original, short communication, case reports, clinical trial, systematic, review, correspondece, Research Letters, etc.

 Research trends. It would be interesting to know how these trends vary between Latin America. Europe and Asia.

**Implications for practice**

 I suggest organizing the published studies: case series, observational study, clinical trials and systematic review, diagnostic test, etc. Although there are very few clinical trials and systematic reviews on sporotrichosis, this evidence should be presented in tables. Furthermore, studies not related to the clinical practice of sporotrichosis should be excluded.

 Finally, sporotrichosis is not limited to dermatology.

**Discussion**

 In the “Discussion” section I would have wished to see more information on implicance practic .

 Limitations should be described and discussed such as: the number of databases included in the study, the studies were not classified, to differentiate the type of scientific evidence for clinical practice, etc.

Is the work clearly and accurately presented and does it cite the current literature?

Yes

If applicable, is the statistical analysis and its interpretation appropriate?

Yes

Are all the source data underlying the results available to ensure full reproducibility?

No

Is the study design appropriate and is the work technically sound?

Partly

Are the conclusions drawn adequately supported by the results?

Partly

Are sufficient details of methods and analysis provided to allow replication by others?

Yes

Reviewer Expertise:

Medical Mycology

I confirm that I have read this submission and believe that I have an appropriate level of expertise to confirm that it is of an acceptable scientific standard, however I have significant reservations, as outlined above.

# Response

**Published:** 2021-03-29

**Authors:** Rodrigo Almeida-Paes

**Affiliations:** Fundacao Oswaldo Cruz, Brazil

We would like to thank this reviewer for his thoughtful comments. Our point-by-point response is presented below:

**Reviewer comment: **Methods. The literature search is limited to two databases. Fig. 1. Present a flow, and follow the guidelines of a scoping review. MedLine / Pubmed and Abstracts of Scientific Conference should be reviewed. There are several published studies on sporotrichosis in journals indexed in SCIELO, LiLACS, etc. These papers have not been included in the study.

**Our response: **The authors used a bibliometric analysis approach to observe the evolution of the world scientific literature and to identify research trends in the sporotrichosis field, focusing on the Brazilian scenario. The authors did not intend to assess the state of the art in the area, but rather to perform a bibliometric analysis with the research effort, which differs from a scoping review.

 The authors chose as methodological approach not to use regional bases such as SciELO and LILACS, due to the difficulty to obtain total equivalence for a global analysis, since other regional bases should be included, making data harmonization difficult.

 Scopus includes a more expanded spectrum of journals (covers 100% of Medline and Embase) and Web of Science covers the oldest publications. Also, metadata for multiple affiliations for each author or contributor are included in Pubmed only at the end of 2014.

 The authors understand the relevance and challenges of integrating different databases in bibliometric analysis to provide a reliable research panorama. The inclusion of limitations will clarify any methodological bias.

**Action taken: **We included a paragraph in the discussion addressing this limitation of study.

**Reviewer comment: **I don’t think that this article contains enough robust data to evidence the implications for practice. The strategy and organization of the information about Implications for practice is not appropriate. A set of words related to tentative language is biased. Furthermore, only the abstracts have been reviewed. To evaluate the implications in practice, it is necessary to organize the different types of published studies: case series, observational study, clinical trials and systematic review, diagnostic test, etc. Furthermore, studies not related to the clinical practice of sporotrichosis should be excluded.

**Our response and action taken: **The text was amended to describe in the methodology the recovery of recommendation messages for practice, research and public health in abstracts based on a wording recovery. Several authors have already demonstrated the importance of abstracts, since they are a condensed version of a scientific research and the most often read section of a research article. 

 Searching abstracts are convenient because they contain most of the article's relevant keywords and appear to be a useful tool to inform to practitioners as a resource for guiding clinical decisions.

 The tentative set of words was already validated by Lynn J et al 2011, where research articles from three leading clinical journals ( *N Engl J Med, JAMA, and Ann Intern Med*) were examined to identify terms that articulated implications (eg, words such as ‘may’, ‘speculate’, ‘suggest’, ‘reinforce’ and ‘potentially’).

**Reviewer comment: **Paragraph 1. I suggest include in Results section include the statistical test.

**Our response and action taken: **This information was included.

**Reviewer comment:** Table 3. I suggest to make a comparison between Brazil, USA, Mexico, China, or hyperendymic areas of sporotrichosis. Scientific production by hyperendemic areas of sporotrichosis should be presented.

**Our response and action taken: **Table 2 was replaced for an improved version as suggested.

 To address the reviewer’s suggestion, the authors included the proportion of publications compared to the total number of articles for each country in the table 1, which shows Top 20 most active countries in the *Sporothrix*/sporotrichosis field according to author’s country of professional affiliation what allows comparison between hyperendemic countries.

**Reviewer comment: **The authors should include the different types of published articles: review, original, short communication, case reports, clinical trial, systematic, review, correspondece, Research Letters, etc.

**Our response: **The sentence in methods was altered in order to clarify the point raised by the reviewer identifying the different types of articles analyzed (original articles, reviews, letters and editorials).

**Action taken: **See Souce of data section.

**Reviewer comment: **Research trends. It would be interesting to know how these trends vary between Latin America. Europe and Asia.

**Our response: **Thank you for this suggestion. The authors agree that this analysis would show a bigger picture. However, for a robust and reproducible analysis, the quantity of publications must be consistent to construct a map based on a co-occurrence matrix. To assess how these trends vary between Latin America, Europe and Asia, the small size of text corpus of each region may not be able, unfortunately, to represent nuances in the research scenario.

**Reviewer comment: **Implications for practice: I suggest organizing the published studies: case series, observational study, clinical trials and systematic review, diagnostic test, etc. Although there are very few clinical trials and systematic reviews on sporotrichosis, this evidence should be presented in tables. Furthermore, studies not related to the clinical practice of sporotrichosis should be excluded.

**Our response: **The text was amended to describe the methodology to recovery recommendations messages for practice, research and public health in abstracts based on wording recovery, rather than a comprehensive search for recommendations for practice. Our original intention with this research was to evaluate a methodology to identify messages and technical recommendations for action in the scientific publications on sporotrichosis. In this first moment, the authors do not intend to analyze the messages, but the validation of the recovery methodology. Further analysis with a broader sample of medical journals to examine how researchers report evidence for action, aiming to assess how they framing their research findings to address implications for practice and public health is needed.

**Action taken: **Changes in the title, abstract (methods section), introduction (last paragraph), results (statements about practice section), and discussion

**Reviewer comment: **Finally, sporotrichosis is not limited to dermatology.

**Our response and action taken: **Thank you for the reminder. The authors were refering to journals with the scopus in dermatology. The text was amended to: “for instance, four of the top ten journals are directed to medical doctors with interest in dermatology”.

**Reviewer comment: **Discussion: In the “Discussion” section I would have wished to see more information on implicance practic. Limitations should be described and discussed such as: the number of databases included in the study, the studies were not classified, to differentiate the type of scientific evidence for clinical practice, etc.

**Our response and action taken: **Thank you for this observation. To clarify the limitations the text was amended and a limitations section was included in the end of the discussion. To address the reviewer’s suggestion, a paragraph describing the applicability of the methodology proposed to the retrieval of the key messages was also included in the discussion section.
